# Editorial: Mechanisms of Ischemia-Reperfusion Injury in Animal Models and Clinical Conditions: Current Concepts of Pharmacological Strategies

**DOI:** 10.3389/fphys.2022.880543

**Published:** 2022-03-21

**Authors:** Rodrigo L. Castillo, Alejandro González-Candia, Rodrigo Carrasco

**Affiliations:** ^1^ Department of Internal Medicine, East Division, Faculty of Medicine, University of Chile, Santiago, Chile; ^2^ Critical Patient Unit, Hospital Salvador, Santiago, Chile; ^3^ Institute of Health Sciences, University O’Higgins, Rancagua, Chile; ^4^ Peter Munk Cardiac Centre, University Health Network, Toronto, ON, Canada

**Keywords:** ischemia-reperfusion injury, cardiomyocytes, blood-brain barrier, oxidative stress, tissue engineering

Ischemia-reperfusion (IR) injury is defined as the paradoxical worsening of cellular dysfunction and death following the restoration of blood flow to previously ischaemic tissues. The restorations of blood flow are essential to salvage ischaemic tissues. However, reperfusion itself causes further damage contributing to reversible and irreversible changes in tissue viability and organ function, the basic pathophysiology of IR injury, especially oxidative stress, and cell death mechanism. When the blood supply is re-established, local inflammation and oxidative stress production increase, leading to secondary injury. Cell damage induced by prolonged IR injury may lead to apoptosis, autophagy, necrosis, and necroptosis. It occurs in a wide range of organs system, including the heart, lung, kidney, and brain. It may involve not only the ischaemic organ itself but may also induce systemic damage to distant organs, potentially leading to multi-system organ failure, as different animal models have shown. Similar responses are seen in clinical patients exposed to acute and chronic IR, but the intensity of the physiological response would determine the activated cellular mechanisms. However, short-term and long-lasting effects are still unclear.

Pharmacological strategies have been proven to be reproducible in preclinical studies across a range of studies with *in vitro* and *ex vivo* experimental models. However, novel pharmacological approaches should be further tested *in vitro* studies as referred to in the articles published in this Research Topic (Flores-Vegara et al., and Chi et al.). This type of approach allows defining the pathophysiological mechanisms and a design towards a more precise clinical trial as developed by translational medicine.

Regarding tissue engineering in hypoxic conditions, described by Zamorano et al., this technique offers a promising toolset to tackle ischemia-reperfusion injuries. It devises tissue-mimetics by using the following principles: 1) the unique therapeutic features of stem cells, i.e., self-renewal, differentiability, anti-inflammatory, and immunosuppressants effects; 2) directed growth factors to drive cell growth and development; 3) functional biomaterials, to provide defined microarchitecture for cell-cell interactions; 4) bioprocess design tools to emulate the macroscopic environment that interacts with tissues. This strategy allows cell therapeutics to address ischemia-reperfusion injury (IRI) in a specific model of clinical settings. It is clearly, Frontier knowledge.

Cerebrovascular permeability is a significant factor determining the cause, progression, outcome, and therapeutic effectiveness of different neurological impairments in postnatal life. In one report, the authors showed that hypoxia and epigenetic modification regulated fetal brain circulation during gestation. In the last decade, several epigenetic mechanisms activated by intrauterine hypoxia have been proposed to regulate the postnatal BBB permeability (Herrera and González-Candia et al.). However, advances in understanding how gestational hypoxia induces Research Topic in the expression of proteins involved in the integrity of the cerebrovascular network remain widely unexplored. On the other hand, another study investigated how Rapalink-1 (mTOR inhibitor) affects neuronal survival and BBB disruption in the cerebral ischemia-reperfusion model within the time window of thrombolysis therapy (Chi et al.) The data demonstrated that inhibiting both mTORC1 and mTORC2 by Rapalink-1 could worsen the neuronal damage in the early stage of cerebral ischemia-reperfusion. The aggravation of BBB disruption could be one of the contributing factors, demonstrating the importance of BBB integrity in brain parenchymal homeostasis.

Communication between cells is a foundational concept for understanding the physiology and pathology of biological systems. Paracrine/autocrine signaling, direct cell-to-cell interplay, and extracellular matrix interactions are three types of cell communication that regulate responses to different stimuli. In the heart, cardiomyocytes, fibroblasts, and endothelial cells interact to form the cardiac tissue. Under pathological conditions, such as myocardial infarction, humoral factors released by these cells may induce tissue damage or protection, depending on the type and concentration of molecules secreted. Cardiac remodeling is also mediated by the factors secreted by cardiomyocytes and fibroblasts that are involved in the extensive reciprocal interactions between these cells. The review of Flores-Vergara et al., shows principal molecules, pathways, and mechanisms underlying cardiomyocyte and cardiac fibroblast crosstalk during ischemia/reperfusion injury. Also, these mechanisms discussed can support the bases of cardioprotective approaches in clinical settings of myocardial IR and secondary remodeling.

We believe that these issues beyond the basic clinical discussion support the search for a novel paradigm for strategies and pharmacological targets currently under development. In addition, these topics are of broad interest to basic science researchers, such as physiology to applied pharmacology.

